# Agmatine Alleviates Cisplatin-Induced Ototoxicity by Activating PI3K/AKT Signaling Pathway

**DOI:** 10.1523/ENEURO.0434-21.2022

**Published:** 2022-03-23

**Authors:** Ying Zhang, Zhe Lv, Qiang He

**Affiliations:** Department of Otolaryngology, the Second Hospital of Hebei Medical University, Shijiazhuang, Hebei 050000, China

**Keywords:** agmatine, cisplatin-induced ototoxicity, PI3K/AKT

## Abstract

Cisplatin-induced ototoxicity can be partially attributed to excessive reactive oxygen species (ROS) production, and agmatine is well-known for the activation of the phosphoinositide-3-kinase (PI3K)/protein kinase B (AKT) pathway to inhibit ROS production. Whether agmatine could be used to alleviate cisplatin-induced ototoxicity is investigated. Cisplatin-exposed House Ear Institute-Organ of Corti 1 (HEI-OC1) cells and cochlear explants showed increased ROS production detected by 2′,7′-dichlorodihydrofluorescein diacetate (DCFH-DA) staining and decreased cell viability detected by Cell Counting Kit-8 (CCK-8) or Myosin 7a staining, which could be reversed by the agmatine pretreatment. Cisplatin intraperitoneally injected C57BL/6 mice demonstrated damaged auditory function as indicated by distortion products otoacoustic emissions (DPOAEs) and auditory brainstem response (ABR) assays, and trans-tympanically administrated agmatine in the left ears could partly prevent the auditory function loss. Mechanistically, downregulated B-cell lymphoma 2 (Bcl-2) expression, upregulated Bcl2-associated x (Bax) expression, and diminished p-PI3K and p-AKT expression were detected in cisplatin-exposed HEI-OC1 cells and cochlear explants, which could be prevented by the pretreatment with agmatine. Our investigation demonstrates that agmatine pretreatment could alleviate cisplatin-induced ototoxicity with the activation of PI3K/AKT signaling pathway.

## Significance Statement

The study demonstrated that agmatine pretreatment could activate phosphoinositide-3-kinase (PI3K)/protein kinase B (AKT) signaling pathway to alleviate cisplatin-induced ototoxicity.

## Introduction

Cisplatin was approved in 1978 by the United States Food and Drug Administration (FDA) to treat ovarian cancer and metastatic testicular patients (Farrell, 2015). Although serious side effects such as bone marrow depression, nephrotoxicity, and ototoxicity can occur, cisplatin remains the most widely used and available chemotherapeutic drug to treat solid malignant tumors (Glynne-Jones and Hoskin, 2007; Chang and Chinosornvatana, 2010; Johnstone et al., 2016; Rottenberg et al., 2021). The incidence of cisplatin-induced ototoxicity can range from twenty percent to seventy percent, which may manifest with progressive, irreversible, and bilateral hearing loss (Tang et al., 2021). Young children are more inclined to cisplatin-induced ototoxicity with delayed speech development and psychosocial and cognitive development (Knight et al., 2005; Rybak et al., 2019).

It is generally believed that cisplatin-induced ototoxicity may be attributed to the excessive reactive oxygen species (ROS) production by the cochlea (Yu et al., 2020), and endoplasmic reticulum stress is a target for treatment of hearing loss (Wang and Xu, 2020). Multiple promising strategies have been performed to alleviate, treat, and prevent cisplatin-induced ototoxicity, and none of these strategies has been confirmed or recommended by the FDA (Gentilin et al., 2019; Mukherjea et al., 2020).

Among the multiple signaling pathways contributing to the survival and differentiation of hair cells, phosphoinositide-3-kinase (PI3K)/protein kinase B (AKT) pathway is well investigated (Liu et al., 2021), which might play an essential role in inner ear hair cells survival to resistance against harmful stimuli (He et al., 2021). It is worth noting that, in neonatal cochlear spiral ganglion explants, PI3K/AKT signaling mediates brain-derived neurotrophic factor-induced neurite formation (Mullen et al., 2012). In the noise-induced cochlea injury, PI3K/AKT pathway activation induced by deferoxamine may promote mesenchymal stem cell homing (Peyvandi et al., 2018).

Agmatine is formed by L-arginine decarboxylation and hydrolyzation to putrescine, which can bind to NMDA receptors and α2-adrenergic receptors to function as novel neurotransmitters and neuromodulators (Regunathan, 2006; Piletz et al., 2013). Substantial preclinical and initial clinical evidence has indicated the possibility to treat opioid addiction, mood disorders, neurotrauma, neurodegenerative diseases, and cognitive disorders (Xu et al., 2018; Akasaka and Fujiwara, 2020).

This investigation utilizes agmatine to treat cisplatin-exposed House Ear Institute-Organ of Corti 1 (HEI-OC1) cells, cochlear explants, or cisplatin affected mice and finds that agmatine alleviates hearing loss with reduced ROS production and cell loss and upregulated PI3K/AKT signal pathway. Therefore, as an adjuvant drug, agmatine has the potential value in reducing ototoxicity caused by cisplatin chemotherapy.

## Materials and Methods

### Cell viability

HEI-OC1 cells (5000/well) were seeded in 96-well plates in three replicates, and relevant agmatine (10, 50, 100, 200 μm) and or cisplatin (5, 10, 30, 50 μm) were incubated for indicated hours. Cell Counting Kit-8 (CCK-8; Dojindo Laboratories) was added to each well with the final concentration of 10% for 4 h. The optical density values were measured at 450 nm with a Bio-Rad plate reader.

### Cisplatin-exposed HEI-OC1 cells culture

HEI‐OC1 cell line was pretreated with 100 μm agmatine for 2 h and then cotreated with 30 μm cisplatin for 24 h in appropriate conditions (33°C, 5% CO_2_, high‐glucose DMEM, 5% fetal bovine serum, Invitrogen).

### ROS detection

2′,7′-dichlorodihydrofluorescein diacetate (DCFH-DA) working solution (10 μm, Beyotime, S0033) was added into six-well plates and incubated the plates at 37°C for 30 min. After the incubation, the fluorescence was observed with the LEXT OLS5100 laser scanning confocal microscope.

### Cisplatin-exposed cochlear explants

Cochleae from C57BL/6 mice (3 d postnatal) were dissected out and seeded intact on Cell-Tak (BD Biosciences) coated glass coverslips, which were further incubated with DMEM/F12 medium supplemented with 1× N2/B27 as recommended by the manufacturer (Invitrogen) at 37°C with 5% CO_2_. Agmatine (100 μm) was used to pretreat cochlear explants for 2 h, and then cisplatin (30 μm) was added to induce the ototoxicity for 24 h.

### Western blotting

HEI-OC1 or cochleae explant lysates were separated by 12% SDS-polyacrylamide gel and transferred to polyvinylidene fluoride membranes, which was further blocked with 5% nonfat dry milk and incubated with primary antibodies against GAPDH, Bcl2-associated x (Bax), BclII, p-PI3K, p-AKT, PI3K, and AKT (Santa Cruz). Peroxidase-conjugated secondary antibody (Sigma-Aldrich, 1:1000 dilution, 2 h, at room temperature) was added, and an ECL system (Sigma-Aldrich) was used to obtain the signal. The intensity of protein bands was quantified with ImageJ software. GAPDH was used as the loading control to normalize the relative expression.

### Cisplatin-exposed mice

C57BL/6 male mice (four-week-old) purchased from Peking Vital River Laboratory Animal Ltd. were maintained. Agmatine (10 μm, 5 μl) was trans-tympanically injected into the left ears, while the same volume PBS was injected into the contralateral ears. Then cisplatin (30 mg/kg) was intraperitoneally administered 2 h later. Seven days after cisplatin administration, the auditory brainstem responses (ABRs) and distortion product otoacoustic emission (DPOAE) measurements were done. All the procedure was approved by the Ethics Committee of the Second Hospital of Hebei Medical University.

### ABR test

ABR assessment was performed as previously reported (McLean et al., 2021). Briefly, anesthetized mice (25 mg/kg xylazine sodium and 100 mg/kg ketamine, i.p.) were kept warm during the ABR recordings process (highest intensity of acoustic stimuli, 90 dB SPL; decrements, 5 dB SPL) at 38°C on the thermostatic heating pad. The hearing threshold at five frequencies (4, 8, 16, 24, and 32 kHz) was detected with TDT System III apparatus (Tucker Davies Technologies).

### DPOAE test

DPOAE was performed as previously reported with a TDT-RZ6 system (Tucker-Davis Technologies; Li et al., 2021). Two sine wave tones with different frequencies but equal intensities (F2 = 1.2F1, F2 ranging from 4 to 40 kHz) were used with 1 s duration to elicit DPOAE. Twenty adjacent frequency bins around the distortion product frequency were averaged as the surrounding noise floor. DPOAE threshold was determined when the signal was over 5 dB SPL and over 2 SDs above the surrounding noise floor.

### Immunofluorescence

Intact cochleae were separated from the temporal bone, which were embedded in Optimal Cutting Temperature O.C.T. medium (Richard-Allan Scientific), snap frozen in liquid nitrogen, and stored at −80°C until use. Five-micrometer sections were cut by a cryostat (Microm HM525). After being fixed with 4% paraformaldehyde for 10 min, the sections were blocked with 10% normal goat serum and permeabilized with 0.3% Triton X-100 for 2 h at room temperature. The Fast ImmunoCytoChemistry Staining kit (Protein Biotechnologies), anti-Myosin 7a antibody (Proteus Bioscience, 25-6790), and DAPI were used for hair cell detection.

### Statistical analysis

The difference between groups was assessed using one or two-way ANOVA analysis before corresponding *post hoc* tests. The significance level was set as *p*-value < 0.05. All statistical analyses were performed with GraphPad Prism (GraphPad Software).

## Results

### Agmatine alleviates cisplatin-induced ototoxicity in HEI-OC1 cells

**Figure 1.**
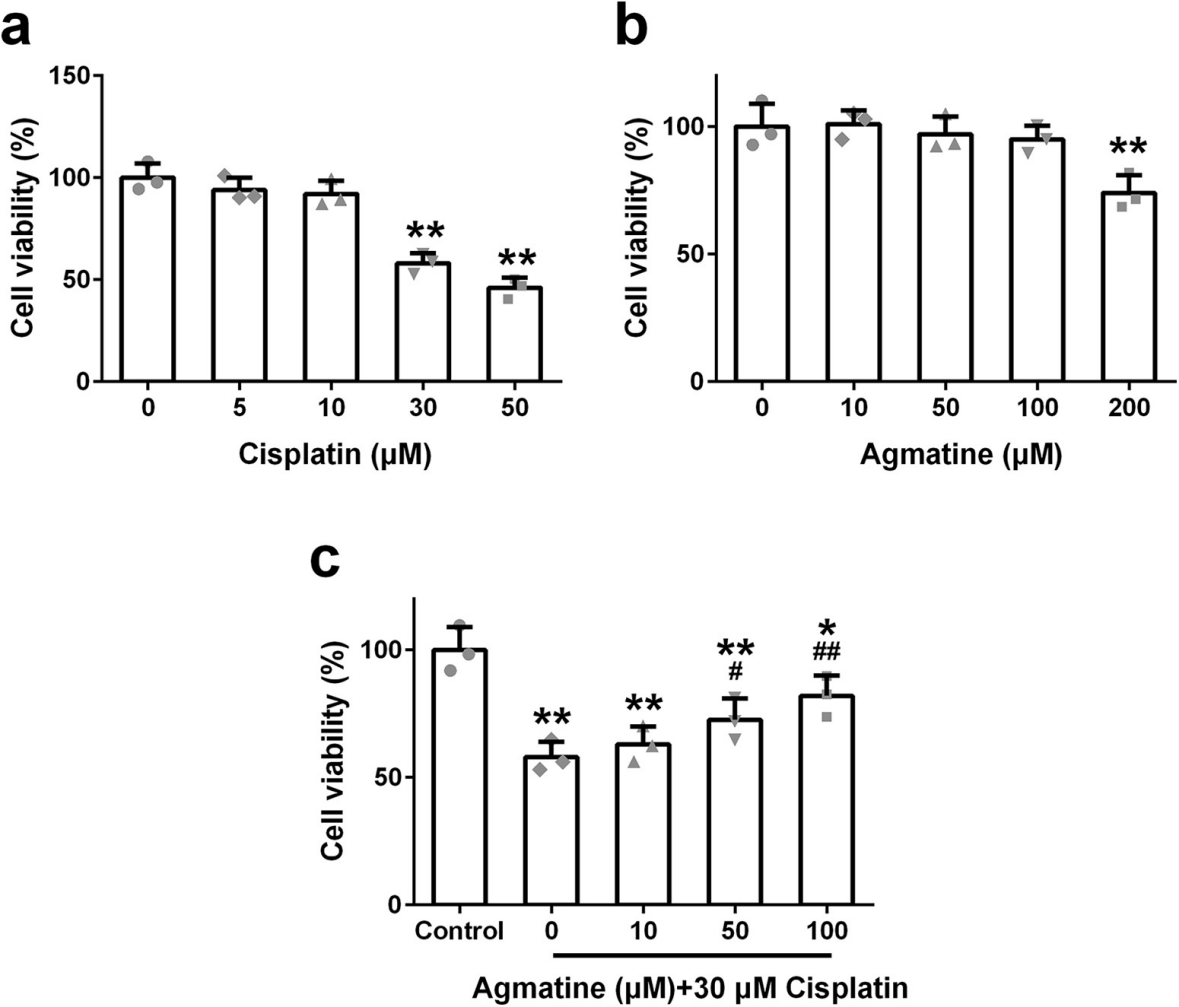
The viability of HEI-OC1 cells with the treatment of designed concentration of cisplatin (***a***), agmatine (***b***), and different concentration of agmatine and 30 μm cisplatin (***c***) for 24 h. Data were represented as mean ± SD, *n* = 3; *p <* *0.05, **p <* *0.01 compared with control group; #*p *<* *0.05, ##*p *<* *0.01 compared with 30 μm cisplatin only group.

To optimize the dose of cisplatin, different concentrations of cisplatin (0, 5, 10, 30, or 50 μm) were used to treat HEI‐OC1 cells for 24 h, and the cell viability was analyzed by CCK‐8 assay. Cisplatin (at a dose >30 μm) can markedly reduce cell viability (Fig. 1*a*). While as to agmatine, only 200 μm agmatine could diminish the viability of HEI-OC1 cells (Fig. 1*b*), which indicated that the dose under 200 μm was safe. To determine the protective effect of agmatine on cisplatin-induced ototoxicity, HEI-OC1 cells were pretreated with different concentrations of agmatine for 2 h and then cotreated with 30 μm cisplatin for 24 h. A significant dose-dependent protective effect was observed, and 100 μm agmatine showed the maximal protective effect (Fig. 1*c*). These results testified that agmatine could protect HEI‐OC1 cells viability on cisplatin exposure, and 100 μm agmatine and 30 μm cisplatin were chosen in the following experiment.

### Agmatine alleviates cisplatin-induced ROS in HEI-OC1 cells

**Figure 2.**
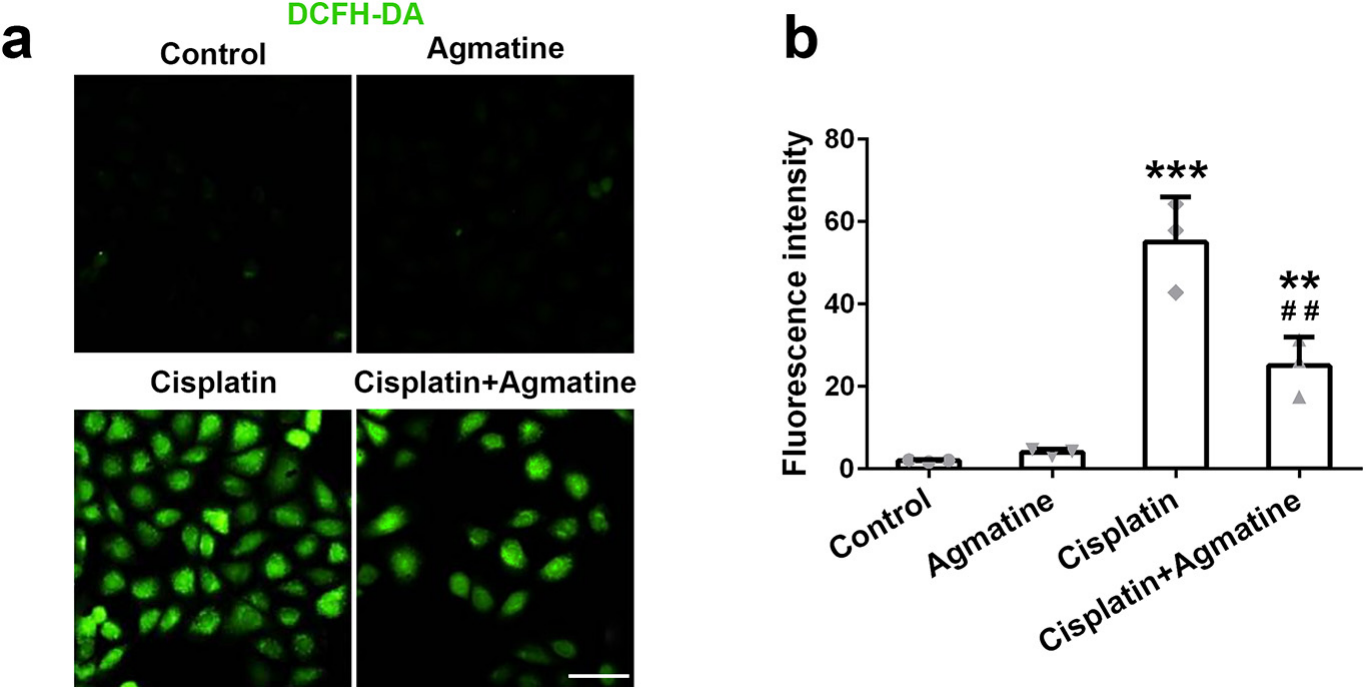
The intracellular level of ROS in HEI-OC1 cells was detected with DCFH-DA staining. ***a***, Fluorescent images from different groups. ***b***, Fluorescence intensity was measured with ImageJ software. Data were represented as mean ± SD, *n* = 3; **p <* *0.01, ***p <* *0.001 compared with control group; ##*p *<* *0.01 compared with cisplatin group. Scale bar: 50 μm.

We examined ROS production with a mitochondria-specific ROS indicator, DCFH-DA, to determine whether agmatine could alleviate cellular oxidative stress. Cisplatin-induced upregulated ROS production, while the pretreatment with agmatine could diminish ROS induction (Fig. 2*a*,*b*). It was worth noting that agmatine alone could not induce the production of ROS. All of these indicated the protection effect caused by agmatine.

### Agmatine alleviates cisplatin-induced cochleae cell apoptosis *in vitro* with upregulated PI3K/AKT pathway

**Figure 3.**
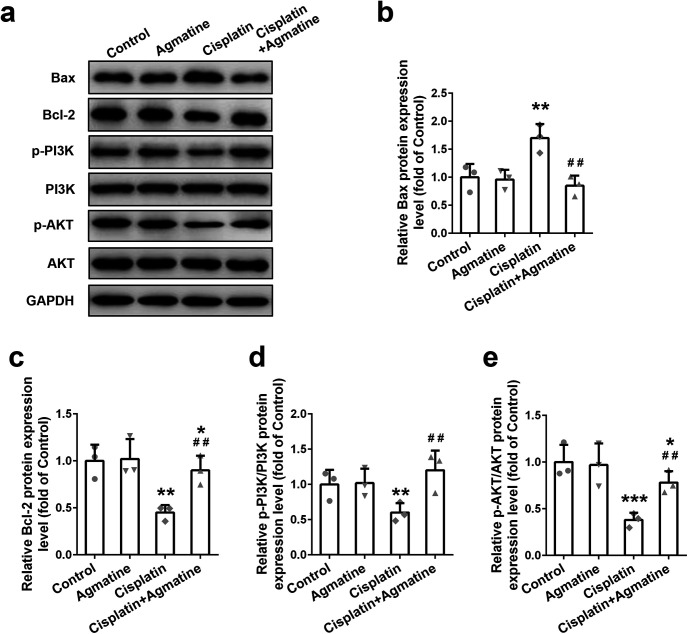
Effect of agmatine on the Bcl-2 family proteins expressions and PI3K/AKT signaling activation in HEI-OC1 cells. Representative Western blot images (***a***) and quantitative analysis of Bax (***b***), Bcl-2 (***c***), p-PI3K (***d***), and p-AKT (***e***). Data were represented as mean ± SD, *n* = 3; *p <* *0.05, **p <* *0.01, ***p <* *0.001 compared with control group; ##*p* < 0.01 compared with cisplatin group.

In order to decipher the relevant pathway for apoptosis, Bcl‐2 family proteins expression was detected with Western blotting in cisplatin-exposed cells. Elevated Bax (pro‐apoptotic; Fig. 3*a*,*b*) and decreased B-cell lymphoma 2 (Bcl-2; anti‐apoptotic; Fig. 3*a*,*c*) were observed, which could be reversed by the pretreatment of agmatine. At the same time, the upstream PI3K/AKT signaling pathway molecules were detected. After cisplatin treatment, downregulated p-PI3K (Fig. 3*a*,*d*) and p-AKT expression (Fig. 3*a*,*e*) were detected. As expected, agmatine administration could upregulate the PI3K/AKT pathway as indicated by the upregulation of p-PI3K and p-AKT. These data demonstrated that agmatine had the ability to inhibit cisplatin-induced apoptosis with upregulated PI3K/AKT signaling pathway in HEI‐OC1 cells.

### Agmatine alleviates cisplatin-induced cochleae explants apoptosis

**Figure 4.**
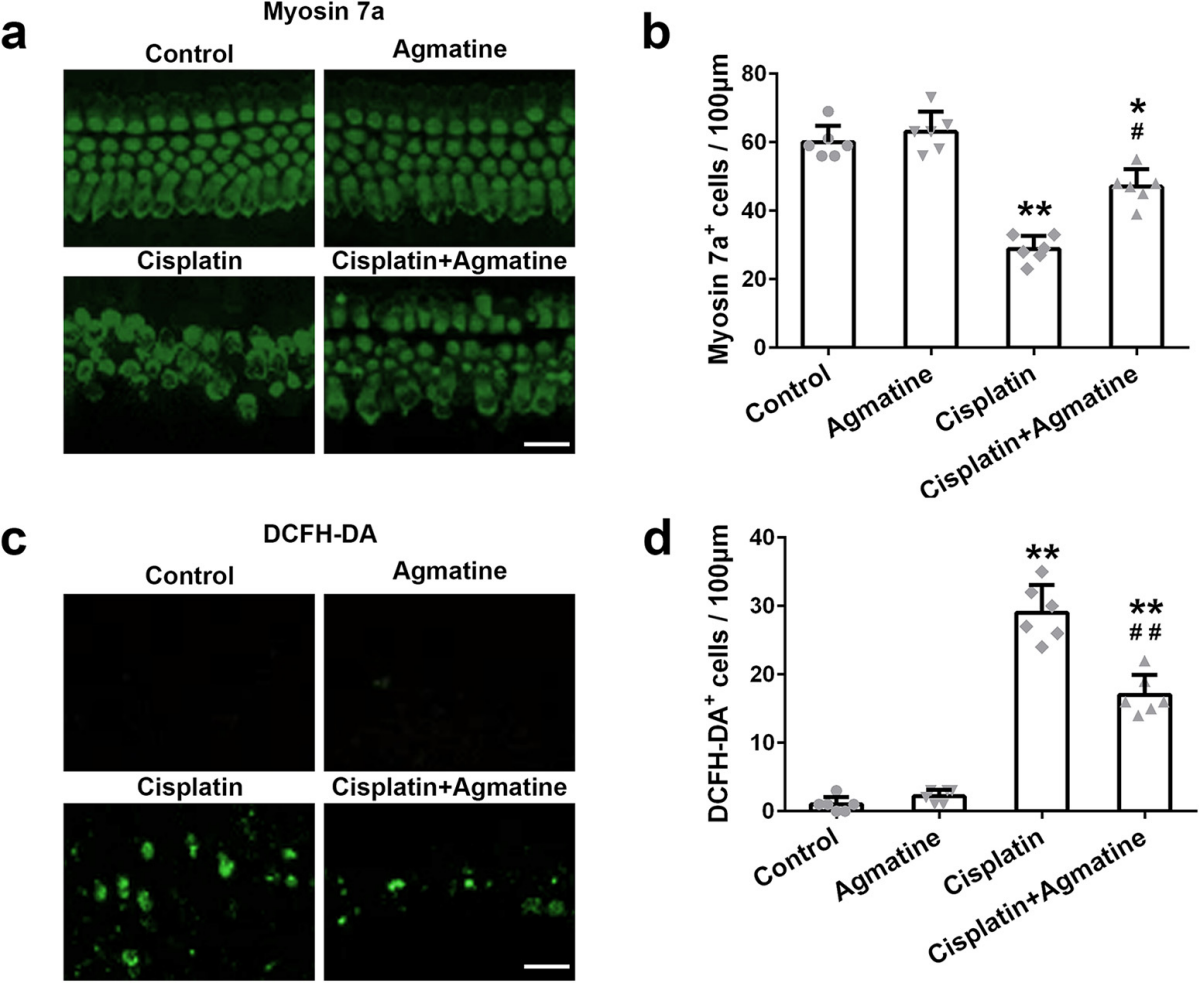
Effects of agmatine on cisplatin-induced hair cell loss (***a***) and ROS level (***c***) in the middle turns of the cochleae explant. Quantification of myosin 7a-positive (***b***) and DCFH-DA-positive (***d***) hair cells. Scale bar: 20 μm. Data were represented as mean ± SD, *n* = 6 for each group; *p <* *0.05, **p <* *0.01 compared with control group; #*p *<* *0.05, ##*p *<* *0.01 compared with cisplatin group.

Myosin 7a staining showed that cisplatin treatment could lead to the conspicuous loss of mature hair cells in the apex (data not shown), basal turns (data not shown), and the middle turn of cochlea. The most significant damage effect was observed on the middle turn of the cochlea, which could be alleviated by the pretreatment of agmatine (Fig. 4*a*,*b*). Accumulation of ROS may lead to the apoptosis of hair cells. No DCFH-DA-positive cells were detected in the untreated or the agmatine-treated cochlear explants (Fig. 4*c*). While significantly increased DCFH-DA-positive cells were detected after cisplatin exposure, this increase was reversed by agmatine pretreatment (Fig. 4*c*,*d*).

### Agmatine stimulates PI3K/AKT signaling to inhibit the apoptosis in cochleae explant induced by cisplatin exposure

**Figure 5.**
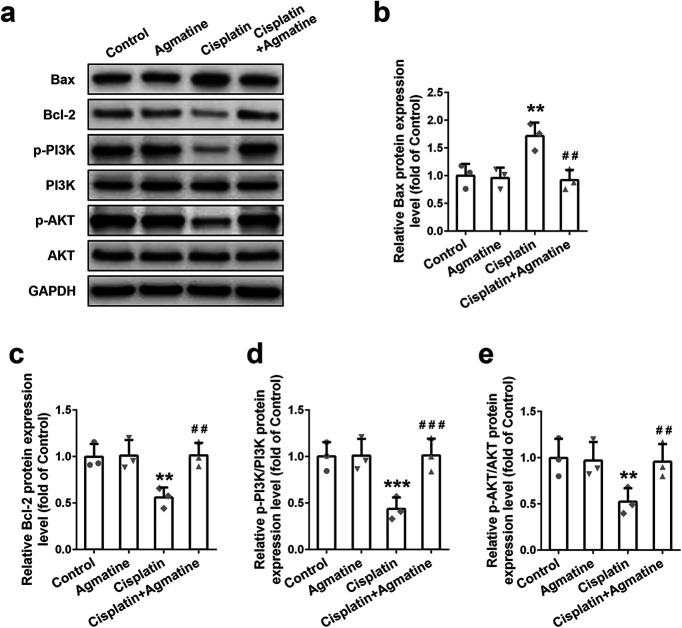
Effect of agmatine on the Bcl-2 family proteins expression and PI3K/AKT signaling pathway activation in the middle turns of the cochleae explant. Representative Western blot images (***a***) and quantitative analysis of Bax (***b***), Bcl-2 (***c***), p-PI3K (***d***), and p-AKT (***e***). Data were represented as mean ± SD, *n* = 3; **p <* *0.01, ***p <* *0.001 compared with control group; ##*p *<* *0.01, ###*p *<* *0.001 compared with cisplatin group.

Cochleae explant was further used to confirm the apoptosis induced by cisplatin exposure. Increased Bax expression (pro‐apoptotic; Fig. 5*a*,*b*) and decreased Bcl-2 expression (anti‐apoptotic; Fig. 5*a*,*c*) were observed in cisplatin-exposed cochleae explant, which could be reversed by the agmatine pretreatment. At the same time, downregulated p-PI3K (Fig. 5*a*,*d*) and p-AKT expression (Fig. 5*a*,*e*) were detected after cisplatin treatment. As expected, agmatine administration could upregulate the PI3K/AKT pathway. All of these data confirmed that agmatine could stimulate PI3K/AKT signaling pathway to inhibit cisplatin-induced intrinsic apoptosis pathway in cochleae explant and HEI‐OC1 cells.

### Agmatine prevents auditory function loss in cisplatin-exposed mice

**Figure 6.**
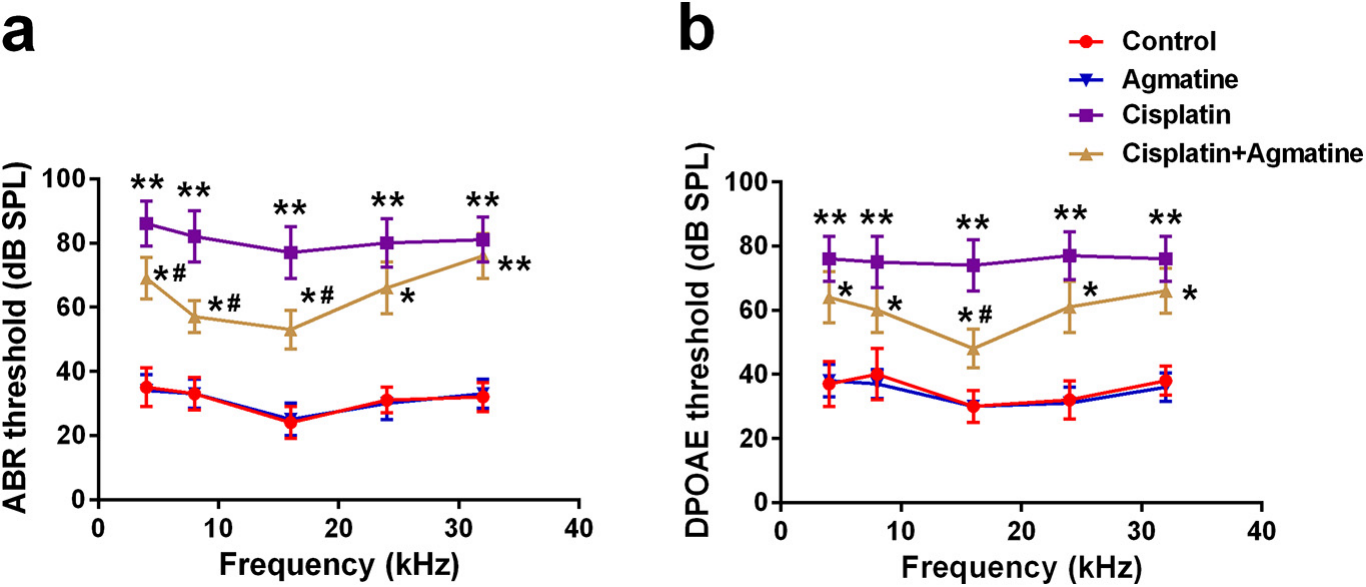
Agmatine prevents auditory function loss in cisplatin-exposed mice. ABR (***a***) and DPOAE (***b***) thresholds were analyzed. Data were represented as mean ± SD, *n* = 6 for each group; *p <* *0.05, **p <* *0.01 compared with control group; #*p *<* *0.05 compared with cisplatin group.

ABR and DPOAE measurements were used to indicate the auditory function. Hearing thresholds were significantly elevated at all frequencies tested 14 d after cisplatin exposure, whereas pretreatment with agmatine could diminish the thresholds (Fig. 6*a*,*b*). All of these indicated that agmatine could partially prevent auditory function loss in cisplatin-exposed mice.

## Discussion

In order to minimize cisplatin-induced ototoxicity, it is vital to find an appropriate strategy to prevent auditory function loss or restore auditory function (Tang et al., 2021). Cisplatin-exposed HEI-OC1 cells, cochleae explant, and mice are used in this investigation, and we confirm that agmatine alleviates cisplatin-induced ototoxicity with upregulated PI3K/AKT signaling pathway. Agmatine supplement may help to reduce cisplatin-induced ototoxicity in clinic.

Cisplatin can transport into cochlea cells and retain for months to years to undergo hydrolysis to form highly reactive aqua cisplatin complexes, which can induce hair cells apoptosis, inflammation, and permanent hearing loss (Rybak et al., 2019). Our results demonstrate the beneficial effect of agmatine pretreatment on cisplatin-induced cochleae cells apoptosis with the inhibition of the downstream mitochondrial apoptotic pathway, thereby protecting cochleae cells from cisplatin-induced ototoxicity in the acute phase. A long-term effect of agmatine administration should be performed in future investigations.

Cisplatin-induced ototoxicity usually appears in the early stages after exposure to cisplatin, primarily affecting the high frequencies and leading to progressive, permanent, and cumulative hearing loss. As indicated in previous reports, DPOAE (Breglio et al., 2017) and ABR (Rybak et al., 2000) are dysregulated in cisplatin-affected mice. Our investigation testifies that agmatine could improve the degenerative auditory responses ranging from 4 and 32 kHz.

Multiple intracellular signaling pathways, including PI3K/AKT pathway, can phosphorylate Bad (serine-136) to inhibit apoptosis (Wang and Youle, 2009; Lu and Imlay, 2021; Muri and Kopf, 2021). Our present study demonstrates that agmatine could increase phosphorylation of PI3K and AKT diminished by cisplatin exposure. The activation of PI3K/AKT may contribute to the anti-ototoxicity effect of agmatine on cisplatin exposure.

The antioxidant effect of agmatine may act as a scavenger against ROS in human neuronal-like SH-SY5Y cells to maintain mitochondrial membrane potential (Condello et al., 2011). In RAW 264.7 cells, agmatine has antioxidant activity against lipopolysaccharides-induced ROS accumulation via the activation of PI3K/Akt pathway (Chai et al., 2016). It is worth noting that agmatine has anti-inflammatory effects, effectively inhibiting the transcription factor NF-κB (Li et al., 2020). As to the safe dose identified in our study (200 μm agmatine), a preprint paper indicates that the safe dose of agmatine can reach to 10 mm (Park et al., 2020). Such discrepancy may need further detailed analysis.

In summation, we demonstrate that agmatine significantly affects the protection against cisplatin-induced ototoxicity by inhibiting ROS production and mitochondrial apoptosis. Our findings further indicate that agmatine can function as a therapeutic or preventive agent in cisplatin-induced ototoxicity.

In conclusion, agmatine can be used to alleviate cisplatin-induced ototoxicity with upregulated PI3K/AKT signaling.

## Synthesis

Reviewing Editor: Christine Portfors, Washington State University Decisions are customarily a result of the Reviewing Editor and the peer reviewers coming together and discussing their recommendations until a consensus is reached. When revisions are invited, a fact-based synthesis statement explaining their decision and outlining what is needed to prepare a revision will be listed below. The following reviewer(s) agreed to reveal their identity: Allison Coffin.

The reviewers agree that the study addresses an important problem and that the data are interesting. The writing however distracts from the impact of the study and in many places is confusing and lacking logical flow. Specifically, the results would benefit from reorganization to improve the logical flow of the manuscript. Some concerns over the ABR data should also be addressed. Overall, major editing of the text and figures will improve the manuscript.

Reviewer 1:

The methods state that ABR responses were measured at 4 frequencies (10, 20, 30, and 40 kHz), but the data in Figure 4 clearly show 5 frequencies, which look to be 4, 8, 16, 24, and 32 kHz). Also, I would expect control C57/B6 mice at this age to have lower auditory thresholds at 8 and 16 kHz, leading to the classic U-shaped audiogram. These data reduce confidence in their ABR recordings.

While the results are interesting, the writing is challenging to follow. In particular, the writing would benefit from clearer rationale for the project as a whole and for specific experiments, as well as clear transitions between sentences. For example, the abstract does a good job of laying out what experiments were performed, but not why agamatine is interesting, or of explaining the overall purpose of the study. The abstract also moves from cell lines and explants, to in vivo experiments, then back to cell lines and explants, making it difficult for the reader to synthesize the information.

For the methods section, I recommend re-ordering to group information by experimental preparation. For example, I think it would help to group all of the cell culture experiment together. That would also help the reader to understand when the relevant assays (cell viability or ROS) were used relative to when the cells were treated with cisplatin and/or agmatine. Also, in the methods, it’s not clear if the western blots were performed on HEI-OC1 cells, cochlear explants, or cochleae from the in vivo experiment. Were the cochleae from the in vivo experiment assessed for hair cell damage?

Similarly to my previous comment, I also recommend reordering the results section to move from HEI-OC1 cells, to the explants, then to the in vivo data. I think this order will increase the logical flow of the manuscript.

There are also grammatical issues that detract from the overall meaning, and some words that appear inaccurate. For example, lines 13-14 say that “damaged auditory function...could be restored with agmatine pre-treatment.” Restoration implies that damage already occurred, e.g., hearing regeneration. I believe “prevented” would be a better term. I do not provide a complete list here of wording concerns, but rather provide a few examples to illustrate some specific concerns.

Minor points:

• Lines 29-30 state that cisplatin-induced toxicities could limit clinical application. However, cisplatin has broad therapeutic use, as stated on lines 28-29, so the statement of limited application does not seem accurate.

• Its unclear what is meant by lines 37-38 regarding interference with anti-tumor activity. I think this comment also applies to lines 164-165. I’m not aware of research showing that all (or even most) possible therapies for cisplatin otoprotection compromise anti-tumor efficacy. Please cite appropriate literature for these statements.

• The introduction would benefit from some information on PI3K/Akt signaling in the context of cisplatin ototoxicity to help the reader understand why this pathway is important, and information on why agmatine may regulate PI3K/Akt signaling.

• Methods. Why only use 30 μM cisplatin for the cell culture experiments? Similarly, why use 30 μM cisplatin for the cochlear explants, and why 30 mg/kg for in vivo cisplatin? Why trans-tympanic administration of agmatine? Please justify based on the literature.

• For lines 110-111, I believe the authors mean that agmatine concentrations under 200 μM did not reduce cell viability. The wording “only a dose up to 200 μM” implies that all doses of 200 μM or less caused damage. Also, this experiment does not measure ototoxicity exactly, but rather viability of HEI-OC1 cells.

• Lines 134-135 (and lines 153-154), I disagree that these data example the “cisplatin-induced intrinsic pathway of apoptosis.” Cisplatin can damage auditory cells via several different mechanisms, including DNA damage, as well as pathways examined here (e.g., oxidative stress). Also, the PI3K/Akt pathway regulates cell protection - I wouldn’t consider it part of a canonical cell death pathway.

• Lines 167-168. I agree that the data provide evidence that agmatine activates PI3K/Akt signaling. However, I disagree that their data show that agmatine confers protection by activating the PI3K/Akt pathway. Studies with PI3K/Akt inhibitors would be needed to support this conclusion. Similarly, lines 174-175 mention that “agmatine can stabilize the mitochondria.” Again, I agree that their data with Bax/Bcl2 protein expression indicate that agmatine can modulate mitochondrial cell death pathways. However, I did not see data that examined mitochondrial stabilization. I suggest modifying their wording to better reflect their findings.

• Lines 170-171, I’m confused about how the cisplatin retention time in the cochlea is relevant for their discussion. Please clarify.

Reviewer 2:

a few comments that need authors’ consideration are as below:

1)Line 32-33 (Introduction section): To support the statement made by authors, the following reference might be cited: Knight et al, 2005; Journal of Clinical Oncology

2)In the Methods section:

a)Were mice of either genders, or only one particular gender got exposed to cisplatin?

b)What was the concentration(s) of N2/B27 in the culture medium used for maintaining cochlear explants; concentration of CCK-8 and DCFH-DA working solution used for cell viability assay and ROS detection, respectively? Did the cisplatin exposed cochlear explants get co-treated with agmatine, if so please mention in the methods section?

c)What was the highest intensity of acoustic stimuli delivered during ABR measurement and the decrements (e.g. 5dB SPL or 10dB SPL) of intensity?

d)Briefly explain the myosin7a staining protocol used for hair cell detection.

3)In the present study, there was reduced cell viability (Figure 1b) observed when 200μM agmatine was used on HEI-OC1 cell line. A recent study demonstrated that the agmatine used was NOT toxic in this cell line at even as high concentration as 10mM (Park et al, 2020; BioRxiv). What could possibly be the reason for the reduced cell viability in the present study? The authors may want to discuss this recent study (Park et al, 2020; BioRxiv) in the discussion section of the manuscript.

4)The length of scale bar (Figure 2a) in the Figure 2 legend needs mention. Also, DCFH-DA may be mentioned in the top penal of Figure 2a for readers ease.

5)The hearing thresholds increased significantly in cisplatin alone exposed mice compared to control (Figure 4). The authors described that this increased thresholds could be diminished (line 139) and restored auditory function (line 140) when mice get intratympanic agmatine treatment prior to cisplatin exposure. However, it appears that the hearing thresholds of these agmatine and cisplatin co-treated mice is still significantly higher than control (Figure 4). This suggests that there was a partial recovery of hearing function rather than full rescue of hearing. Did the authors look at synapses in cochlear tissues from these mice? Also, was there any sign of gender specific differences in hearing threshold recovery, in case mice from both genders were included in this study? The authors are encouraged to provide timelines and age of the mice when the ABR and DPOAE measurements were done.

6)In the figure 5a&b, the hair cells were quantified from middle turn of the cochlea to determine hair cell loss. Did the author look at the apex or basal turns of the cochlea?

7)The authors are encouraged to specify the method used to determine the relative expression levels of different proteins in the Western blot (Fig 3 and 6). Was there any software used to compare the relative band intensities?
